# Stroke Strikes in Bangladesh: Current Insights and Future Directions

**DOI:** 10.7759/cureus.37882

**Published:** 2023-04-20

**Authors:** Arefin Sadat, Vivek Podder, Rakesh Biswas

**Affiliations:** 1 Medicine, Chandpur Medical College and Hospital, Chandpur, BGD; 2 General Medicine, Tairunnessa Memorial Medical College and Hospital, Gazipur, BGD; 3 Public Health, University of Adelaide, Adelaide, AUS; 4 Medicine, Kamineni Institute of Medical Sciences, Hyderabad, IND

**Keywords:** emergency medical services, stroke risk factor, stroke condition in bangladesh, cerebrovascular disease, global healthcare systems

## Abstract

Stroke is a neurological condition attributable to vascular injury (e.g., infarction, hemorrhage) of the central nervous system. Globally, it ranks high among the leading causes of death. The poor stroke management system in Bangladesh is contributing to the country’s rapid rise in stroke incidence. Stroke-related mortality and disability can be decreased by being aware of and taking steps to address potential risk factors. The population in this area has a generally poor understanding of strokes. Important avenues for preventing stroke in this population may include an effective public awareness campaign which includes spreading knowledge regarding early signs of stroke (facial drooping, arm weakness, speech difficulties, and time), the golden hour of stroke, cardiopulmonary resuscitation, the development of structured emergency medical care, appropriate rehabilitation, control of blood pressure and blood glucose, and cessation of smoking.

## Editorial

Non-communicable diseases have surpassed infectious diseases and nutritional deficiencies as the leading causes of death worldwide in recent decades. Stroke plays a cogent role in this drastic change. The socioeconomic impact of stroke is significant in both industrialized and non-industrialized nations. Stroke is the major cause of disability in the community and the second biggest cause of mortality worldwide, according to the World Health Organization (WHO), with 1.5 billion people affected annually. Over 5 million people die from strokes and the remaining individuals are paralyzed [1]. The family becomes burdened by these patients. People in their 40s are more likely to suffer a stroke. However, children can experience a stroke as well.

A stroke occurs when a blood vessel occlusion or hemorrhage reduces or interrupts the blood supply to the brain. In consequence, the brain is deprived of sufficient oxygen, and brain cells begin to die. There are two fundamental types of strokes. The first one occurs when a clot blocks blood vessels in the brain, and brain tissue dies from a lack of oxygen. The second is a type of stroke brought on by bleeding from a broken blood artery [2]. Typically, these are the result of aneurysms. A transient ischemic attack (TIA) is an intermittent delay in the passage of blood to the brain. Typically, the clot dissipates on its own or is dislodged, and the duration of the symptoms is shorter than five minutes. A TIA does not cause permanent harm, but it is a *warning stroke* that indicates an impending stroke.

In developing nations, the rates of stroke-related death and disability are astronomically high. Globally, approximately 75% of stroke-related deaths and 81% of stroke-related disabilities occur in developing nations [3]. Both infectious and non-infectious diseases threaten the large and growing population of Bangladesh. After heart disease and infectious diseases such as the flu and pneumonia, stroke is the third biggest cause of death in Bangladesh. According to the most recent WHO data published in 2020, there were 134,166 stroke-related fatalities in Bangladesh, accounting for 18.74% of all deaths. Bangladesh’s age-adjusted death rate of 119.20 per 100,000 population places it 41st globally [4]. Hypertension accounts for 63% of all stroke risk factors, followed by cardiovascular disease (24%), diabetes mellitus (21%), and high cholesterol (7%) [3].

The stroke prevalence in Bangladesh was estimated using data from a population survey that included 15,627 adults aged 40 and above. Stroke prevalence was reported to be 2%, 3%, 0%, and 1% among people aged 40-49, 50-59, 60-69, 70-79, and 80+ years, respectively [5]. High out-of-pocket costs for families, unequal rich-poor access, poor care quality, a paucity of qualified service providers, and a proliferation of unregulated informal providers are only some of the persistent, severe problems plaguing the health systems of Bangladesh. The movement toward universal health care coverage has been impeded because of all these factors. The scarcity of trained neurologists and neuroimaging facilities in Bangladesh also contributes to its high prevalence.

The most effective method for preventing a stroke is to treat its underlying causes. A change in lifestyle, including regular exercise, a balanced diet, maintaining a healthy weight, and abstaining from tobacco and alcohol, can help people achieve this goal. A nutritious diet includes abundant fruits, whole grains, vegetables, nuts, legumes, and seeds. Additionally, people can reduce their risk of stroke by controlling their blood pressure, receiving treatment for heart disease, and maintaining their diabetes.

Rehabilitation is an essential and continuous component of stroke treatment. Recovery from a stroke frequently requires specialized therapies and networks of support, such as speech therapy, physiotherapy, and social assistance. Currently, Bangladesh Rural Advancement Committee (BRAC), a non-profit organization, is working to educate the people of Bangladesh about the warning signs of stroke and provide rehabilitative services to those who cannot afford medical care. The Centre for the Rehabilitation of the Paralysed (CRP) is another organization doing important work in stroke rehabilitation by providing physiotherapy, speech and language therapy, and occupational therapy to stroke patients as well as raising awareness about the signs of stroke in the general population. Even though BRAC and CRP are pioneers in primary stroke countermeasures, the Bangladesh government must prioritize healthcare development to accommodate the country’s growing population and rising stroke incidence.

Stroke care in Bangladesh is very fragile and needs massive improvements. Especially in outlying locations, there needs to be an increase in the number of stroke doctors and rehabilitation facilities. Those who have survived a stroke should have access to more community resources. Prevention programs for strokes must be repeatedly introduced to the mass community. The severity of the stroke in Bangladesh can be reduced by adopting these steps.

The number of strokes in Bangladesh has increased dramatically over the past few decades, and the country’s lack of neurologists and specialized hospitals has contributed to a rise in the severity of the outcomes for those who suffer from strokes. Many stroke risk factors are changeable and susceptible to improvement through lifestyle modification, including physical activity, diet, smoking cessation, and alcohol use. Lifestyle intervention programs and health behavior theories can play a crucial role in enabling the modification of health habits. It is the need of the hour to prioritize the health system considering these issues, as stroke has lasting monetary effects on victims, their families, and society as a whole.
